# Chylous drainage through percutaneous cholecystostomy: an extremely rare complication

**DOI:** 10.1093/jscr/rjae094

**Published:** 2024-02-28

**Authors:** Christina Ellison, Yuichi Igarashi, Noubar Kevorkian

**Affiliations:** General Surgery Residency Program, University of Connecticut, Farmington, CT 06032, USA; University of Connecticut School of Medicine, Farmington, CT 06032, USA; Department of Surgery, The Hospital of Central Connecticut, New Britain, CT 06052, USA

**Keywords:** percutaneous cholecystostomy tube, acute cholecystitis, iatrogenic chyle leak, chylous ascites, chylothorax

## Abstract

Chyle leak is a rare but potentially morbid complication of abdominal surgery. There have been seven reported cases of chylous ascites following cholecystectomy, but no such occurrences are reported with percutaneous cholecystostomy tube (PCT) insertion. We report the case of a 67-year-old female with stage IVb recurrent uterine papillary serous carcinoma and extensive abdominal surgical history including a paraesophageal hernia repair, and a robotic hysterectomy, bilateral salpingo-oophorectomy, pelvic and para-aortic lymphadenectomy, gastrocolic omentectomy, and hepatoduodenal lymphadenectomy. The patient presented with clinical findings suggestive of acute cholecystitis and decision was made to proceed with PCT placement. The PCT was dislodged and replaced during her course and several days after chylous output was noted from the PCT. The remainder of her hospital course was complicated by persistent distributive shock, adrenal insufficiency, and continued chyle leak. She ultimately was transitioned to inpatient hospice and died shortly after.

## Introduction

Benign gallbladder disease is highly prevalent worldwide. Laparoscopic cholecystectomy (LC) remains the gold standard treatment modality with minimal associated morbidity and mortality [1, 2]. In a subset of patients with multiple comorbidities or concurrent acute and critical conditions, acute cholecystitis could be managed with a percutaneous cholecystostomy tube (PCT). PCT serves as a temporizing, sometimes permanent, measure of obtaining source control in patients who are not ideal candidates for general anesthesia or surgical intervention.

Despite abundant literature on common LC complications like bile duct injury and bile leak, reports of chylous leak post-cholecystectomy are scare, with fever than a dozen cases. While spontaneous postoperative chylous ascites has been documented in a handful of cases, there have been no cases reporting this complication post-PCT insertion.

Chylous ascites is a rare but potentially morbid complication that presents with accumulation of lymphatic milky fluid in the peritoneal cavity that is rich in triglycerides and lymphocytes. This condition is often attributed to traumatic abdominal injury or lymphatic obstruction from benign or malignant causes [3, 4]. Since cholecystitis is one of the most common surgical diagnoses, recognizing potential morbid complications of management options is crucial.

## Case description

We report the case of a 67-year-old female with chronic bilateral lower-extremity lymphedema and stage IVb recurrent uterine papillary serous carcinoma who was treated with PCT placement for acute cholecystitis and developed chylous drainage from her PCT.

The patient has an extensive surgical history. At age 62, she underwent a laparoscopic hiatal hernia repair, partial gastrectomy and partial omentectomy for a strangulated paraesophageal hernia. Pathology from this operation revealed metastatic high-grade carcinoma. Subsequently, she underwent neoadjuvant and adjuvant chemotherapy, as well as robotic hysterectomy, bilateral salpingo-oophorectomy, bilateral pelvic and para-aortic lymphadenectomy, gastrocolic omentectomy, tumor debulking, ureteral lysis, and excision of periportal lymph nodes. Two years later, she had radiographic signs of recurrence in peri-aortic and iliac lymph node basins and was maintained on pembrolizumab and lenvima, with reportedly adequate oncologic control.

**Figure 1:**
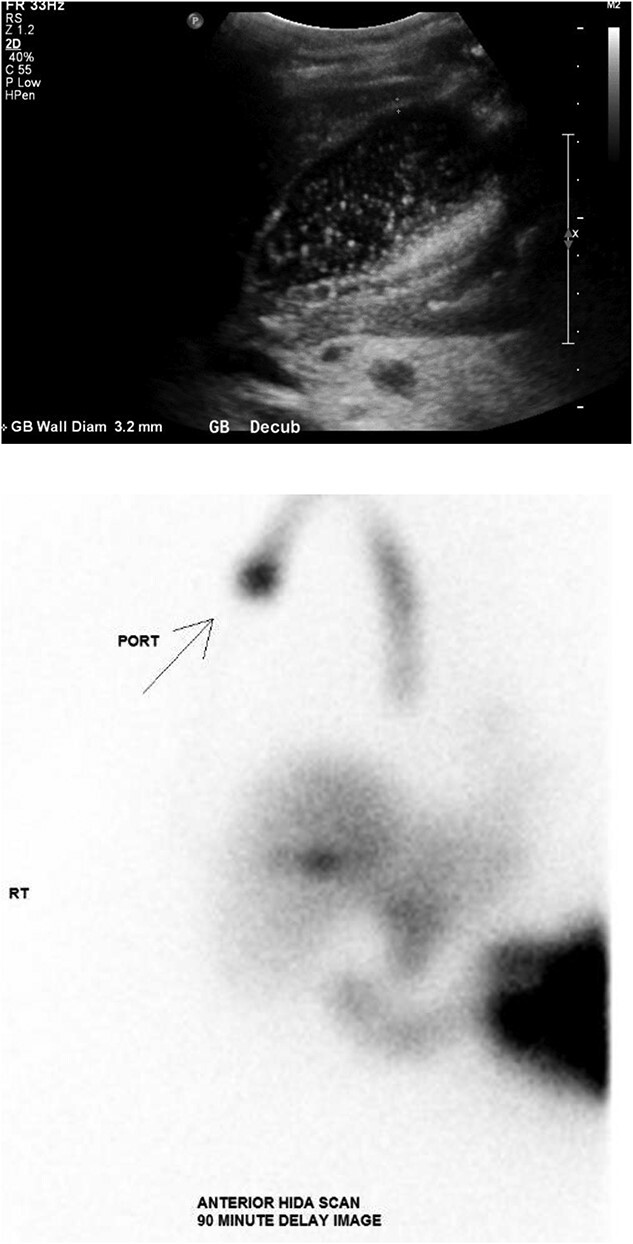
(Top) Ultrasound of the abdomen demonstrating distended gallbladder full of sludge with wall thickness of 3.2 mm. (Bottom) Nuclear medicine hepatobiliary scan demonstrating no gallbladder visualization after 90 minutes strongly supportive of acute cholecystitis.

The patient was presented to the emergency department with a 4-week history of abdominal pain and intractable nausea and vomiting. She was vitally normal with normal liver function tests and no leukocytosis. Imaging revealed significant gallbladder distension, wall thickening, and pericholecystic inflammatory changes suggestive of acute cholecystitis (Fig. 1).

**Figure 2:**
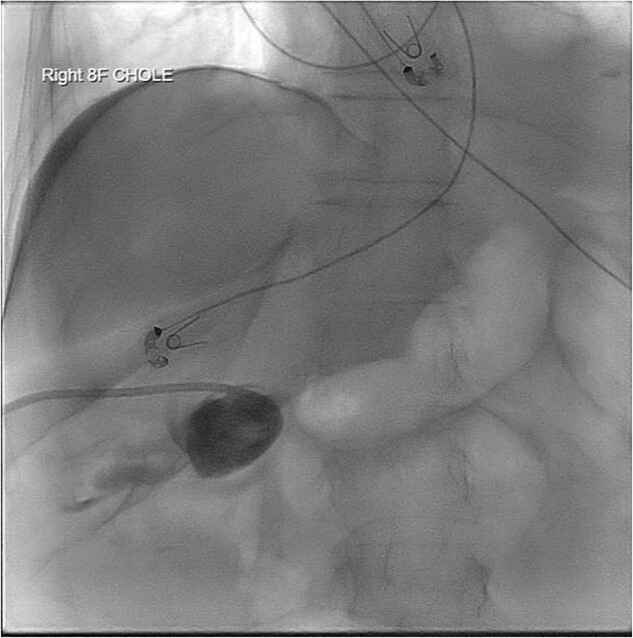
PCT check using fluoroscopy showing retracted tube in the peritoneal fluid.

Given her surgical history, duration of symptoms, and active chemotherapy, she was deemed high-risk for intraoperative biliary injury and poor wound healing. Cholecystectomy was deferred and the gallbladder was decompressed with PCT placement. The patient demonstrated significant symptomatic improvement, diet was advanced, and she was discharged home on hospital Day 5. Several hours after discharge, she returned due to lethargy and was found to be in septic shock. Repeat imaging revealed a dislodged PCT. She was admitted to the surgical intensive care unit (ICU) for vasopressor support and mechanical ventilation. Cholangiogram was performed and revealed the pigtail catheter in the peritoneal space (Fig. 2). Under fluoroscopic guidance, the pigtail catheter was replaced without difficulty. She was started on antibiotics for bacteremia and remained in the ICU for management of septic shock and acute hypoxic respiratory failure.

Five days post-PCT replacement, the drainage became a white thick consistency, with an output of 1200 milliliters (ml) in 24 hours. The triglyceride level of the fluid was 683 mg/dL, consistent with chylous output. After transitioning to parental nutrition, her output decreased to 700 ml in 24 hours. However, this was short-lived and outputs increased to 800–1000 ml per day.

**Figure 3:**
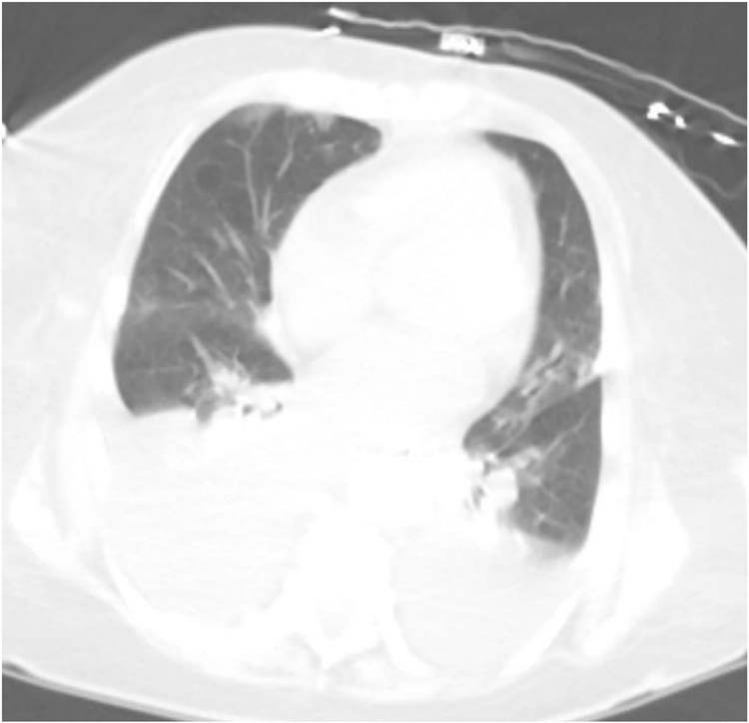
Computed tomography (CT) chest demonstrating bilateral pleural effusions.

**Figure 4:**
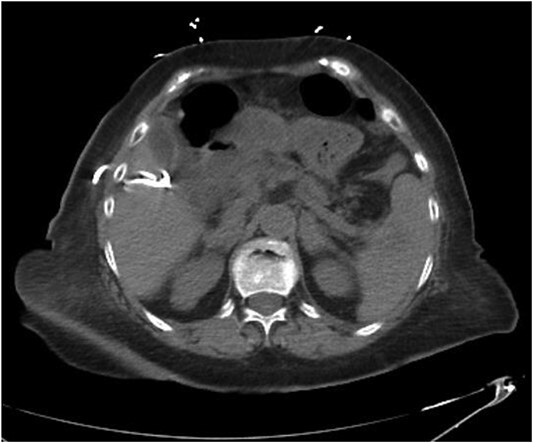
CT abdomen and pelvis demonstrating positioning of PCT.

Repeat imaging was performed and revealed peri-hepatic ascites, significant anasarca and bilateral pleural effusions (Fig. 3) while the PCT remained in good position (Fig. 4). Bilateral chest tube placement and drainage of the pleural effusions revealed chylothorax and malignant cells. Cultures of both PCT output at the time of insertion and pleural fluid cultures grew no organisms.

**Figure 5:**
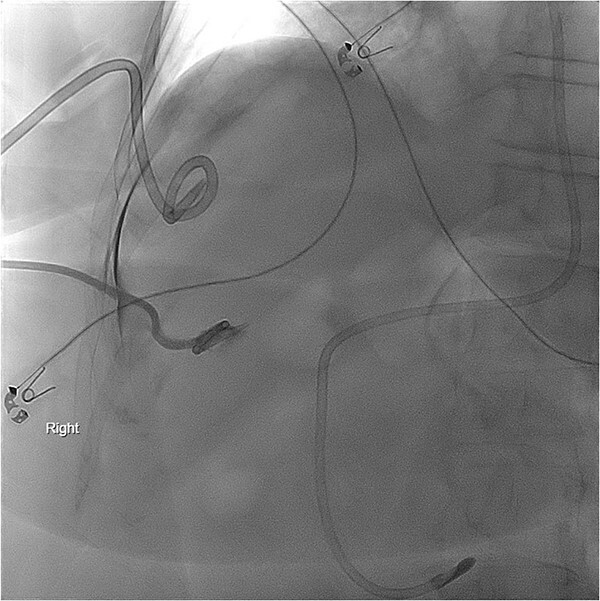
PCT check using fluoroscopy showing the pigtail portion of the cholecystostomy tube within the gallbladder lumen as well as some leakage into the perihepatic space. No opacification of the pleural space.

She was managed with bowel rest, parenteral nutrition, and octreotide. PCT output decreased but remained chylous throughout the hospitalization. Subsequent cholangiogram and PCT check revealed no fistulous connection to the thoracic cavity or lymphatic system (Fig. 5). Her hospitalization was further complicated by persistent distributive shock, adrenal insufficiency, and continued chyle leak. Though she was successfully extubated, her clinical status continued to decline. Per the wishes of the patient and family, she was transitioned to inpatient hospice and died shortly after.

## Discussion

The lymphatic vessels around the gallbladder are part of the hepatic and periportal lymphatic system. These lymphatic vessels drain lymph from the liver, gallbladder, and bile ducts, eventually converging to drain into the thoracic duct [5, 6]. Violation of lymphatics in the area can result in chyle leak and there have been multiple cases of post-LC chyle leaks reported.

Drs Jensen and Weiss (2006) were the first who documented chylous ascites following LC. They described a 31-year-old female who had a percutaneous drain placed 2 weeks after initial surgery, which revealed chylous ascites. Unable to be resolved conservatively, she underwent lymphoscintigraphy localizing the leak in the gallbladder fossa and subsequent laparoscopic oversewing of the leak [7]. Huang *et al*. (2009) and Gogalniceanu *et al*. (2010) both describe cases of chyle leaks following LC for gallstone pancreatitis [3, 8]. Both cases were able to be managed conservatively with diet modification. Bansal *et al*. (2016) reported a 54-year-old female who developed chylous ascites following LC requiring re-laparoscopy and drain placement [4]. Yao *et al*. (2018) reported another case of a chylous leak with high-volume drain outputs that required re-operation and oversewing of the leak within the liver bed [9]. Ong *et al*. (2021) documented a 48-year-old male who had chylous output from his drain in the first postoperative day following uneventful LC with cholangiogram. He had a low-volume leak and was successfully managed with a fat-free diet [6]. Cheng *et al*. (2022) describes another case requiring paracentesis and ultimately lymphoscintigraphy, which identified a leak for which the patient underwent re-exploration and ligation of the lymphatic fistula [10]. Most recently, Philip *et al*. (2023) reported a simultaneous chyle leak and bile leak postoperatively managed by percutaneous drainage, octreotide, and low-fat diet [11].

To our knowledge, this is the first reported case of chyle leak complicating PCT insertion. PCT insertion can theoretically violate the lymphatics either through traumatic insertion, compression and erosion, or infection. Both the initial insertion and the replacement of the PCT were reported to be performed without difficulty. It is possible that injury to the lymphatic system could have occurred after her initial hospital discharge when the tube was found to be dislodged. In the context of this patient with prior complex abdominal surgeries involving lymph node dissection and subsequent cancer recurrence within the lymphatic system, the exact mechanism for the chyle leak is unclear. There are several contributing factors to this patient’s overall risk for injury of the lymphatic system. She underwent tumor debulking and dissection into multiple lymph node basins including the periportal lymph nodes. Additionally, cancer recurrence several years later within the lymphatic system may have resulted in lymphatic obstruction. These factors could have predisposed her to the PCT chyle leak. Whether it was caused by violation of the retroperitoneal lymphatic channels during tube insertion, dislodgement, exchange of the PCT, or potentially erosion of the tube into the nearby hepatic lymphatic channels, could not be clinically determined. This case poses an interesting clinical question as to additional potential risks involved in PCT insertion, particularly in high-risk populations.
